# Supplementary material for: Psychometric Properties of the Coparenting Relationship Scale in Ecuadorian Parents
Source: Eur J Investig Health Psychol Educ. 2025 Jun 25;15(7):117. doi: 10.3390/ejihpe15070117 (PMC12295813; doi:10.3390/ejihpe15070117)
Supplement: Supplementary file 1 [file ejihpe-15-00117-s001.zip › ejihpe-3609468-supplementary.pdf]

Supplementary Information

Table S1 Supplement

Descriptive analysis of the items in the Coparenting Scale.

	Valid	*Missing*	*Median*	*Mean*	*Std. Deviation*	*Skewness*	*Std. Error of Skewness*	*Kurtosis*	*Std. Error of Kurtosis*	*Shapiro-Wilk*	*P-value of Shapiro–Wilke*	*Minimum*	*Maximum*	*25th percentile*	*75th percentile*
I1	867	0	5.000	4.881	1.555	-1.598	0.083	1.798	0.166	0.730	< .001	0	6	5.000	6.000
I2	867	0	5.000	4.088	2.003	-0.837	0.083	-0.561	0.166	0.832	< .001	0	6	3.000	6.000
I3	866	1	5.000	4.401	1.919	-1.157	0.083	0.117	0.166	0.783	< .001	0	6	3.000	6.000
I4	867	0	5.000	4.588	1.741	-1.279	0.083	0.673	0.166	0.782	< .001	0	6	4.000	6.000
I5	867	0	3.000	2.953	2.001	-0.002	0.083	-1.271	0.166	0.916	< .001	0	6	1.000	5.000
I6	867	0	5.000	4.705	1.816	-1.462	0.083	1.030	0.166	0.725	< .001	0	6	4.000	6.000
I7	867	0	4.000	3.412	2.299	-0.274	0.083	-1.493	0.166	0.849	< .001	0	6	1.000	6.000
I8	867	0	1.000	2.090	2.139	0.583	0.083	-1.116	0.166	0.836	< .001	0	6	0.000	4.000
I9	867	0	3.000	3.158	2.112	-0.119	0.083	-1.399	0.166	0.895	< .001	0	6	1.000	5.000
I10	867	0	5.000	4.195	1.901	-0.915	0.083	-0.344	0.166	0.834	< .001	0	6	3.000	6.000
I11	867	0	3.000	3.203	2.092	-0.136	0.083	-1.354	0.166	0.900	< .001	0	6	1.000	5.000
I12	867	0	1.000	1.644	1.924	0.974	0.083	-0.326	0.166	0.803	< .001	0	6	0.000	3.000
I13	866	1	0.000	1.493	1.987	1.105	0.083	-0.203	0.166	0.748	< .001	0	6	0.000	3.000
I14	867	0	5.000	4.336	1.849	-1.018	0.083	-0.090	0.166	0.820	< .001	0	6	3.000	6.000
I15	867	0	3.000	3.032	2.030	-0.018	0.083	-1.317	0.166	0.911	< .001	0	6	1.000	5.000
I16	866	1	1.000	1.850	2.088	0.762	0.083	-0.867	0.166	0.808	< .001	0	6	0.000	3.000
I17	867	0	5.000	4.183	2.070	-0.933	0.083	-0.508	0.166	0.799	< .001	0	6	3.000	6.000
I18	867	0	5.000	4.235	1.820	-0.889	0.083	-0.282	0.166	0.847	< .001	0	6	3.000	6.000
I19	867	0	5.000	4.379	1.923	-1.117	0.083	0.019	0.166	0.789	< .001	0	6	3.000	6.000
I20	867	0	5.000	3.691	2.480	-0.466	0.083	-1.503	0.166	0.776	< .001	0	6	1.000	6.000
I21	867	0	0.000	1.176	1.810	1.480	0.083	0.919	0.166	0.688	< .001	0	6	0.000	2.000
I22	867	0	0.000	1.285	1.892	1.376	0.083	0.565	0.166	0.706	< .001	0	6	0.000	2.000
I23	867	0	5.000	4.315	2.020	-1.017	0.083	-0.326	0.166	0.785	< .001	0	6	3.000	6.000
I24	867	0	5.000	4.595	1.879	-1.330	0.083	0.595	0.166	0.745	< .001	0	6	4.000	6.000
I25	867	0	5.000	4.494	1.821	-1.220	0.083	0.418	0.166	0.786	< .001	0	6	4.000	6.000
I26	867	0	5.000	4.288	1.945	-1.045	0.083	-0.152	0.166	0.802	< .001	0	6	3.000	6.000
I27	867	0	5.000	4.323	1.944	-1.064	0.083	-0.086	0.166	0.799	< .001	0	6	3.000	6.000
I28	867	0	4.000	3.368	2.221	-0.230	0.083	-1.458	0.166	0.868	< .001	0	6	1.000	6.000
I29	867	0	4.000	3.563	2.191	-0.373	0.083	-1.334	0.166	0.863	< .001	0	6	1.000	6.000
I30	867	0	5.000	4.534	1.951	-1.258	0.083	0.277	0.166	0.743	< .001	0	6	4.000	6.000
I31	153	714	2.000	2.373	2.109	0.509	0.196	-1.078	0.390	0.870	< .001	0	6	0.000	4.000
I32	151	716	1.000	1.762	2.035	0.887	0.197	-0.651	0.392	0.798	< .001	0	6	0.000	3.000
I33	152	715	0.000	1.033	1.588	1.725	0.197	2.026	0.391	0.684	< .001	0	6	0.000	1.000
I34	151	716	0.000	1.132	1.660	1.603	0.197	1.524	0.392	0.705	< .001	0	6	0.000	1.000
I35	152	715	0.000	1.211	1.646	1.309	0.197	0.536	0.391	0.742	< .001	0	6	0.000	2.000

Note. Mardia's multivariate normality test yielded significant results for skewness. These results indicate that the data do not follow a multivariate normal distribution, as the p-value is less than .05, leading to the rejection of the null hypothesis of normality. Bartlett's test was statistically significant, $\chi 2(595)$=20100.360, p<.001, indicating that the correlation matrix is not an identity matrix. This suggests that the variables are sufficiently correlated to proceed with factor analysis.

Table S2. Supplementary information.

Reliability and validity (convergent and discriminant) of the gender invariance of Model 6 with three factors (22 items).

	Group		Factor 1	Factor 2	Factor 3	ω	α	ω Total	α Total
Configural	Woman	Factor 1	0.565*			0.795	0.794	0.906	0.838
		Factor 2	**0.112****	0.641*		0.958	0.957		
		Factor 3	**0.417****	**0.237****	0.478*	0.813	0.848		
	Man	Factor 1	0.564*			0.794	0.795	0.881	0.749
		Factor 2	**0.065****	0.556*		0.935	0.939		
		Factor 3	**0.406****	**0.338****	0.527*	0.864	0.866		
Metric	Woman	Factor 1	0.563*			0.794	0.794	0.916	0.838
		Factor 2	**0.112****	0.642*		0.958	0.957		
		Factor 3	**0.417****	**0.237****	0.481*	0.835	0.848		
	Man	Factor 1	0.565*			0.796	0.795	0.871	0.749
		Factor 2	**0.065****	0.542*		0.924	0.939		
		Factor 3	**0.406****	**0.338****	0.514*	0.847	0.866		
Scalar	Woman	Factor 1	0.563*			0.794	0.794	0.916	0.838
		Factor 2	**0.112****	0.642*		0.958	0.957		
		Factor 3	**0.417****	**0.237****	0.481*	0.835	0.848		
	Man	Factor 1	0.565*			0.796	0.795	0.871	0.749
		Factor 2	**0.065****	0.541*		0.923	0.939		
		Factor 3	**0.406****	**0.338****	0.514*	0.847	0.866		
Strict	Woman	Factor 1	0.563*			0.794	0.794	0.922	0.838
		Factor 2	**0.112****	0.644*		0.962	0.957		
		Factor 3	**0.417****	**0.237****	0.486*	0.843	0.848		
	Man	Factor 1	0.565*			0.795	0.795	0.855	0.749
		Factor 2	**0.065****	0.535*		0.913	0.939		
		Factor 3	**0.406****	**0.338****	0.505*	0.831	0.866		

Note. Factor 1—shared parenting agreement; Factor 2—closeness, support, and approval of shared parenting; Factor 3—weakness of shared parenting; * AVE—average variance extracted (convergent validity); ** HTMT—heterotrait–monotrait ratio (discriminant validity).

Supplementary Table S1 reported the reliability and validity (convergent and discriminant) of Model 6, which included three factors and 22 items, examining gender invariance for women and men. The factors evaluated were as follows: Factor 1 (agreement in shared parenting); Factor 2 (closeness, support, and approval in shared parenting); and Factor 3 (weakness in shared parenting).

For the women's group, in the configural model, the Omega (ω) and Alpha (α) reliability values showed acceptable consistency, with ω = 0.795 and α = 0.794 for Factor 1; ω = 0.958 and α = 0.957 for Factor 2; and ω = 0.813 and α = 0.848 for Factor 3. In the configural model for men, these values were similar: ω = 0.794 and α = 0.795 for Factor 1; ω = 0.935 and α = 0.939 for Factor 2; and ω = 0.864 and α = 0.866 for Factor 3.

The metric model demonstrated consistent values across both groups, supporting metric invariance for gender. In scalar and strict models, the Omega and Alpha values for the factors also remained stable, reinforcing invariance at these levels. Slight increases were observed in the total reliability coefficients (ω total and α total), especially for women, where total Omega reached a peak of 0.922 in the strict model.

Regarding convergent and discriminant validity, AVE (average variance extracted, denoted with *) and HTMT (heterotrait–monotrait ratio, denoted with **) indicated satisfactory psychometric properties, with AVE exceeding 0.5 for all factors and HTMT below the 0.85 threshold, confirming discriminant validity among the factors.

Table S3. Supplementary information.
Reliability and validity (convergent and discriminant) of the pairwise invariance of Model 6 with three factors (22 items).

Invariance testing	Group		Factor 1	Factor 2	Factor 3	ω	α	ω Total	α Total
Configural	Sin Pareja	Factor 1	0.570*			0.800	0.792	0.928	0.861
		Factor 2	**0.078****	0.588*		0.950	0.947		
		Factor 3	**0.334****	**0.155****	0.456*	0.834	0.820		
	Con pareja	Factor 1	0.561*			0.793	0.793	0.851	0.731
		Factor 2	**0.098****	0.502*		0.925	0.928		
		Factor 3	**0.425****	**0.347****	0.497*	0.849	0.857		
Metric	Sin Pareja	Factor 1	0.563*			0.798	0.792	0.924	0.861
		Factor 2	0.078	0.588*		0.950	0.947		

		Factor 3	0.334	0.155	0.436*	0.825	0.820		
		Factor 1	0.562*			0.794	0.793		
	Con pareja	Factor 2	**0.098****	0.499*		0.923	0.928	0.853	0.731
		Factor 3	**0.425****	**0.347****	0.497*	0.850	0.857		
		Factor 1	0.563*			0.798	0.792		
	Sin Pareja	Factor 2	**0.078****	0.587*		0.950	0.947	0.923	0.861
Scalar		Factor 3	**0.334****	**0.155****	0.436*	0.825	0.820		
		Factor 1	0.562*			0.794	0.793		
	Con pareja	Factor 2	**0.098****	0.499*		0.923	0.928	0.853	0.731
		Factor 3	**0.425****	**0.347****	0.497*	0.849	0.857		
		Factor 1	0.601*			0.851	0.792		
	Sin Pareja	Factor 2	**0.078****	0.619*		1.001	0.947	0.981	0.861
Strict		Factor 3	**0.334****	**0.155****	0.480*	0.908	0.820		
		Factor 1	0.554*			0.783	0.793		
	Con pareja	Factor 2	**0.098****	0.490*		0.907	0.928	0.828	0.731
		Factor 3	**0.425****	**0.347****	0.485*	0.828	0.857		

Note. Factor 1—shared parenting agreement; Factor 2—closeness, support and approval of shared parenting; Factor 3—weakness of shared parenting; * AVE—average variance extracted (convergent validity); ** HTMT—heterotrait–monotrait ratio (discriminant validity).

Table S2 provides insights into the reliability and validity (both convergent and discriminant) of the pairwise invariance for Model 6, which consists of three factors assessed across 22 items. The three factors identified are as follows: (1) shared parenting agreement, (2) closeness, support, and approval of shared parenting, and (3) weakness of shared parenting. In the configural invariance testing, participants without a partner demonstrated good reliability, with Factor 1 showing an Omega (ω) of 0.800 and Cronbach's Alpha (α) of 0.792, while Factor 2 had higher reliability (ω = 0.950, α = 0.947). In contrast, participants with a partner exhibited lower reliability across all factors, particularly for Factor 1 (ω = 0.793, α = 0.793) and Factor 3 (ω = 0.849, α = 0.857). The metrics reveal consistent reliability across both groups, with overall ω and α values of 0.924 and 0.861 for those without a partner and 0.853 and 0.731 for those with a partner, respectively.

Convergent validity, assessed via average variance extracted (AVE), and discriminant validity, measured through the heterotrait–monotrait ratio (HTMT), are confirmed across both configurations, indicating that the factors are both reliable and distinct in the assessment of shared parenting dynamics.

Table S4. Supplementary information.
Reliability and validity (convergent and discriminant) of the Model 6 rupture invariance with three factors (22 items).

Invariance testing	Group		Factor 1	Factor 2	Factor 3	ω	α	ω Total	α Total
Configural	Rupture	Factor 1	0.593*			0.813	0.802	0.907	0.854
		Factor 2	**0.111****	0.624*		0.957	0.954		
		Factor 3	**0.412****	**0.199****	0.470*	0.809	0.830		
	No rupture	Factor 1	0.558*			0.791	0.792	0.872	0.767
		Factor 2	**0.060****	0.525*		0.932	0.934		
		Factor 3	**0.408****	**0.260****	0.492*	0.847	0.855		
Metric	Rupture	Factor 1	0.582*			0.812	0.802	0.920	0.854
		Factor 2	**0.111****	0.624*		0.958	0.954		
		Factor 3	**0.412****	**0.199****	0.453*	0.831	0.830		
	No rupture	Factor 1	0.559*			0.792	0.792	0.871	0.767
		Factor 2	**0.060****	0.523*		0.931	0.934		
		Factor 3	**0.408****	**0.260****	0.491*	0.842	0.855		
Scalar	Rupture	Factor 1	0.582*			0.812	0.802	0.919	0.854
		Factor 2	**0.111****	0.624*		0.958	0.954		
		Factor 3	**0.412****	**0.199****	0.453*	0.831	0.830		
	No rupture	Factor 1	0.559*			0.792	0.792	0.871	0.767
		Factor 2	**0.060****	0.523*		0.931	0.934		
		Factor 3	**0.408****	**0.260****	0.491*	0.841	0.855		
Strict	Rupture	Factor 1	0.605*			0.845	0.802	0.968	0.854
		Factor 2	**0.111****	0.648*		0.996	0.954		
		Factor 3	**0.412****	**0.199****	0.493*	0.904	0.830		
	No rupture	Factor 1	0.555*			0.786	0.792	0.853	0.767
		Factor 2	**0.060****	0.516*		0.918	0.934		
		Factor 3	**0.408****	**0.260****	0.481*	0.825	0.855		

Note. Factor 1—shared parenting agreement; Factor 2—closeness, support and approval of shared parenting; Factor 3—weakness of shared parenting; * AVE—average variance extracted (convergent validity); ** HTMT—heterotrait–monotrait ratio (discriminant validity).

Table S3 presents the reliability and validity (convergent and discriminant) for the invariance testing of Model 6, focusing on three factors across 22 items, in contexts of "rupture" and "no rupture" of relationships. The three factors include (1) agreement on shared parenting, (2) closeness, support, and approval of shared parenting, and (3) weakness in shared parenting. Across the levels of invariance tested—configural, metric, scalar, and strict—Omega (ω) and Cronbach's Alpha (α) coefficients were reported for each factor and the overall model. In the "rupture" group, Factor 1 showed high reliability (ω = 0.813, α = 0.802), with total reliability values (ω total = 0.907; α total = 0.854). Factor 2 had the highest reliability (ω = 0.957, α = 0.954), while Factor 3 was somewhat lower (ω = 0.809, α = 0.830). In the "no rupture" group, reliability was also adequate, but generally lower across factors, with Factor 1 at ω = 0.791 and α = 0.792, and Factor 2 at ω = 0.932 and α = 0.934. AVE (*) values confirm convergent validity, and HTMT (**) ratios support discriminant validity, indicating that the factors are distinct and consistent across groups and levels of invariance.

Figure S1. Supplementary information. Coparenting in the Ecuadorian sample (n = 867).

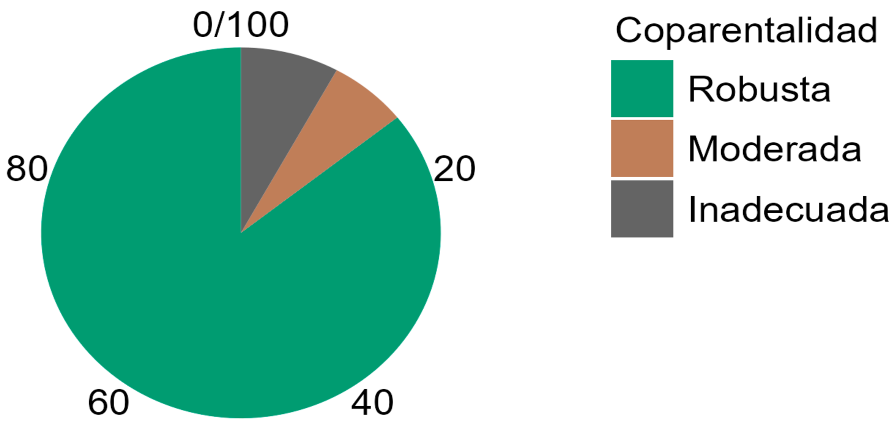
0/100
20
40
60
80
Coparentalidad
Robusta
Moderada
Inadecuada
